# Long-term brain fog and cognitive impairment in previously hospitalized COVID-19 patients

**DOI:** 10.1371/journal.pone.0309102

**Published:** 2024-08-29

**Authors:** Barbara Junco, Daniel Samano Martin Del Campo, Vela Karakeshishyan, Danielle Bass, Evie Sobczak, Emily Swafford, Ana Bolanos, Joshua Rooks, Bernard S. Baumel, Alberto R. Ramos, Tatjana Rundek, Ayham Alkhachroum

**Affiliations:** 1 Department of Neurology, University of Miami Miller School of Medicine, Miami, FL, United States of America; 2 Department of Neurosurgery, University of Miami Miller School of Medicine, Miami, FL, United States of America; Christian Care Thomas Jefferson University, UNITED STATES OF AMERICA

## Abstract

**Objectives:**

Limited research exists on COVID-19 associated brain fog, and on the long-term cognitive and psychiatric sequelae in racially and ethnically diverse patients. We characterize the neuropsychological sequelae of post-acute COVID-19 in a diverse cohort and investigate whether COVID-19 clinical severity remains associated with brain fog and cognitive deficits approximately 2 years post infection.

**Methods:**

A cross-sectional study of patients with a history of COVID-19 hospitalization (March-September 2020). COVID-19 clinical severity was indexed using the National Early Warning Score 2 and a comprehensive neuropsychological tele-battery was administered 2 years post discharge. Pearson’s r correlations assessed association, while independent sample t-tests examined group differences. Significant outcomes were further analyzed using multiple regression and ANCOVAs, adjusting for key covariates.

**Results:**

In 41 adult patients (19 female, 30 Hispanic, 13 Black, mean age of 65 (*SD* = 15), COVID-19 level of severity was associated with greater number of endorsed brain fog symptoms (Pearson’s *r* = .34, 95% CI [.04, .59]), worse overall cognitive functioning (global cognition: r = -.36, 95% CI [-.61, -.05]) and reduced performance on an attention and working memory task (digit span backwards: *r* = -.41, 95% CI [-.66, -.09]) at 2-year follow-up. Brain fog symptoms most associated with COVID-19 severity included difficulty focusing (*r* = .46, 95% CI [.18, .67]), detached (*r* = .41, 95% CI [.12, .64]) and feeling sleepy (*r* = .40, 95% CI [.11, .63]). Patients’ cognitive performance was generally below average (global cognition z-score: *M* = -.96, *SD* = .66), with group differences based on sex and ethnicity evidenced on individual cognitive tests.

**Discussion:**

This study emphasizes the importance of continued research on the long-term effects of COVID-19 infection on neuropsychological outcomes, particularly among underrepresented, health-disparate groups. Greater understanding of these associations could improve detection and treatment of those at increased risk of cognitive decline or impairment.

## Background

COVID-19 continues to affect millions of people globally [1]. While the initial focus of research and treatment was on the acute respiratory and physiological symptoms caused by SARS-CoV-2 (severe acute respiratory syndrome coronavirus 2) , there is growing evidence of the neuropsychological post-acute sequelae of COVID-19 (PASC) [1, 2]. Over 200 million people worldwide continue to experience long-term COVID-19 related symptoms [3], with about 50% of patients exhibiting at least one symptom 12 months after acute infection [4]. Previous studies have documented neuropsychological features of PASC at 4, 6 and 12 months [5–7]. One recent prospective cohort study in Sweden conducted 165 telephone interviews at 4 months post hospital discharge and again at 24 months [8]. Results at 24 months suggested that most patients had decreased quality of life due to ongoing symptoms. Consistent with previous coronavirus outbreaks [9], studies suggest that PASC is associated with persistent neuropsychological and psychiatric issues, especially in individuals hospitalized with severe COVID-19. Additionally, pre-existing comorbidities may become exacerbated in these individuals, quality of life may worsen, and survivors are at increased risk of experiencing worse health outcomes [10]. The persistent neuropsychological and psychiatric effects of PASC, including cognitive impairment, depression, anxiety, and sleeplessness are a growing cause for concern [11]. Subjective cognitive issues, known as “brain fog”, including forgetfulness, difficulty concentrating, and mental fatigue [12], are frequently reported [13]. Many post-acute COVID-19 survivors exhibit measurable difficulties with memory, attention, and executive function [5], however levels of impairment and affected domains remain unclear.

Underrepresented groups in the United States, such as Hispanics, Blacks, and women, may be at a higher risk of PASC [14]. Although further research is needed to identify the mechanisms behind this increased risk, it is likely influenced by disparities in the biological and social determinants of health [15]. Hispanics and Blacks were overwhelmingly affected by acute COVID-19 morbidity and mortality [16]. The impact of PASC on these populations is less well known, but preliminary research suggests they have a higher prevalence of cognitive issues and disproportionate rates of mood disorders [10, 17]. Women are three times more likely to be diagnosed with PASC [18]. Considering these demographic groups remain underrepresented in COVID-19 research and clinical research at large [19, 20], it is critical to accurately characterize the neuropsychological outcomes of PASC and identify associated risk factors.

The focus of this study was to administer a comprehensive neuropsychological tele-battery to evaluate long-term associations between brain fog, objective assessment of cognitive function, and self-reported psychiatric measures of mood and sleep quality in a racially and ethnically diverse cohort. While our primary aim was to characterize the neuropsychological profile of this cohort, we expected that patients who experienced the highest COVID-19 clinical severity while hospitalized would exhibit worse long-term neuropsychological outcomes.

## Methods and materials

### Study setting

This study was conducted at the University of Miami Hospital, an academic medical center and tertiary care hospital.

### Study design

This was an observational, cross-sectional study with a retrospective chart review component.

### Study period

Recruitment for the brain fog and cognition study began on 14 July 2021 and concluded on 30 September 2022.

### Source of population

Patients included in this study were previously hospitalized at the University of Miami Hospital and had to have been already consented into the parent study [21].

### Sample size determination and sampling technique

A total of 411 patients who received a SARS-CoV-2 positive polymerase chain reaction (PCR) test upon admission to hospital during the first two waves of COVID-19 (March 2020 to September 2020) were consented into the parent study. Between hospital admission and this sub study, 129 patients were confirmed deceased. Of the remaining 282 patients screened, 81 did not meet eligibility criteria, 134 did not answer the phone and 26 refused to participate. A total of 41 patients that could be reached, met eligibility, and agreed to participate

### Inclusion and exclusion criteria

Eligibility criteria were determined via review of patient electronic medical records.

Inclusion Criteria: Patients must have been previously consented into the parent study [21] and hospitalized at the University of Miami Hospital with a positive SARS-CoV-2 PCR test.

Exclusion Criteria: Pre-existing comorbidities that could bias results (i.e., dementia or other major neurodegenerative disease, prior cerebrovascular accident, epilepsy or seizures, traumatic brain injury with loss of consciousness, recent cancer treated with chemo or radiation therapy). Non-English and non-Spanish speaking individuals, individuals living in nursing homes or assisted living facilities were also excluded.

### Data collection tools and procedures

Eligible and consenting patients were given a comprehensive neuropsychological telephone battery. Verbal consent was obtained from participants and documented in consent notes by delegated study team members due to pandemic era public health guidelines. The local Institutional Review Board at the University of Miami granted approval (20200404) to the study and approved a waiver of written consent.

. STROBE (Strengthening the Reporting of Observational Studies in Epidemiology) guidelines for observational studies were followed.

### Operational definitions

#### Neuropsychological battery

The neuropsychological battery was given over the phone to assess aspects of cognitive function. It was administered by two fully trained clinical raters who were fluent in the patient’s preferred language of testing (English or Spanish), with an administration time of approximately 35–45 minutes. Patients were instructed to find a quiet and comfortable place in their home, let others in the home know they would be occupied for 45 minutes and not write anything down. Demographic information was also collected (for list see Table 1).

**Table 1:** Sample descriptive statistics.

Total Sample (*N* = 41)	
Sex, *n*(%)	
Female	19 (46.3)
Male	22(53.7)
Ethnicity | Race, *n*(%)	
Hispanic | White	23 (56.1)
Hispanic | Black / Mix	7 (17.1)
Non-Hispanic | White	5 (12.2)
Non-Hispanic | Black / Mix	6 (14.6.)
Age (years), *M*(*SD*)	65.1 (15.1)
Education (years), *M*(*SD*)	14.3 (3.1)
Language of Testing, *n*(%)	
Spanish	25(61.0)
English	16(39.0)
Length of hospitalization (days), *M*(*SD*)	15.3(19.1)
Time Since Discharge (months), *M*(*SD*)	22.2(2.1)
COVID Clinical Severity (NEWS 2), *n*(%)	
Low	6 (14.6)
Medium	18 (43.9)
High	17 (41.5)
Respiratory Distress, *n*(%)	
ARDS/AHRF	23(56.1)
SOB/None	18(43.9)
Comorbidities, *n*(%)	
Hypertension	23 (56.0)
Type 2 Diabetes	16 (39.0)
Hyperlipidemia	9 (22.0)
Asthma	7 (17.1)
COPD	6 (14.6)
Atrial Fibrillation	6 (14.6)
Kidney Disease	6 (14.6)
Coronary Artery Disease	5 (12.2)
Thyroid Disease	5 (12.2)
Obstructive Sleep Apnea	3 (7.3)
Myocardial Infarction	3 (7.3)
Congestive Heart Failure	2 (4.9)
Pulmonary Embolism	2 (4.9)

#### Self-reported brain fog and psychiatric measures

Self-report measures included the Patient Health Questionnaire-9 (PHQ-9) [22], to assess for depression, the Generalized Anxiety Disorder-7 (GAD-7) [23], to assess anxiety, the Pittsburgh Sleep Quality Index (PSQI) [24], to evaluate sleep quality, and a Brain Fog Questionnaire (BFQ) adapted from Ross et al [25], to measure brain fog symptoms. The BFQ consists of 19 items which assess various aspects of brain fog symptoms, such as exhaustion, difficulty with concentration, forgetfulness, mental confusion, and difficulty with word finding. For each item, patients were asked to endorse (“yes” = 1 or “no” = 0) whether they have experienced the given symptom within the past two weeks. Each item on the BFQ was also rated on a Likert scale to gauge both frequency (0 = Never to 7 = All Day) and severity (1 = Mild, 2 = Moderate, 3 = Severe). The total score represents the sum of all 19 endorsed brain fog items and ranges from 0 to 19, with higher scores indicating greater severity. For a description of the self-report psychiatric measures administered to participants, such as the PHQ-9, GAD-7 and PSQI, see the **S1 File**.

#### Standardized cognitive assessments

The comprehensive battery included validated standardized measures of verbal learning and memory, such as the Hopkins Verbal Learning Test–Revised [26, 27], the Controlled Oral Word Association Test letters (FAS letters for English and PTM letters for Spanish speakers) and verbal and category fluency, tests (animals and fruits) [28–31], processing speed and executive set-shifting, were assessed with the Oral Trail Making Test parts A and B (OTMT) [32], and attention and working memory with the Wechsler Adult Intelligence Scale–Fourth Edition (WAIS-IV) Digit Span subtests (forward, backward, sequence) [33, 34]. Normative samples were derived to calculate standardized Z-scores for cognitive measures among both English and Spanish speaking patients. The validity of in-home tele-based administration of these measures has been documented elsewhere [35–37].

#### Variables linked to COVID-19 from admission through hospitalization

Variables included number of days in the hospital, pre-morbid medical diagnoses and diagnoses at discharge, level of respiratory distress including acute respiratory distress syndrome (ARDS), acute hypoxic respiratory failure (AHRF) and shortness of breath (SOB), and type of respiratory support while in the hospital. For calculating COVID-19 clinical severity via the National Early Warning Score 2 (NEWS2) [38], vitals associated with lowest SpO2 (saturation of peripheral oxygen) and hypercapnic status were captured and variables input included respiratory rate, status of hypercapnic respiratory failure (yes/no), room air or supplemental oxygen (yes/no), temperature, systolic blood pressure, pulse, status of consciousness (‘alert’ or ‘new-onset confusion (or disorientation/agitation), responds to voice, responds to pain or unresponsive’). NEWS2 scores are categorized into low (0–4), medium (5–6), and high (≥7) clinical risk.

### Statistical analysis

For our primary outcome, the characterization of brain fog was made via review of descriptive statistics of individual questionnaire symptom items with a specific focus on item prevalence. Pearson’s *r* correlation analyses were conducted to assess the association between BFQ item frequencies with measures of COVID-19 clinical severity (NEWS2), depression (PHQ-9), anxiety (GAD-7) and sleep disturbance (PSQI). Pearson’s *r* correlations were run to assess the association between BFQ total scores with COVID-19 clinical severity, depression, anxiety, sleep disturbance, and cognition as well as demographic variables of age and total comorbidities. Group differences in BFQ total score based on sex, ethnicity, and level of respiratory distress were assessed with independent sample *t*-tests.

For our secondary outcome of cognition, scores were standardized (z-scores) based on previously documented normative samples. An index of global cognitive functioning was calculated as the average of standardized z-scores across all cognitive measures. The characterization of cognitive and psychiatric measures was made via review of descriptive statistics and independent sample *t*-tests to assess differences based on sex and ethnicity.

For our final outcome Pearson’s *r* correlation analyses were run to assess the association between COVID-19 clinical severity (NEWS2) and cognitive and psychiatric outcomes. Independent sample *t*-tests were also run to evaluate group differences based on inpatient diagnosis of acute respiratory distress syndrome (ARDS) or acute hypoxemic respiratory failure (AHRF) versus those with either shortness of breath (SOB) or no respiratory symptoms on physical exam.

Standard multiple regression and analyses of covariance (ANCOVAs) were run to determine whether corresponding significant findings from Pearson’s r correlations and t-tests remained after controlling for relevant covariates (i.e., demographic variables and total number of medical comorbidities, etc.)

## Results

The characteristics of the study sample are shown in Table 1. Among all 41 patients (46% female; age *M* = 65.1, *SD* = 15.1), 25 (61%) were tested in Spanish. Duration of hospitalization ranged from 2 to 96 days (*M* = 15, *SD* = 19) and time from discharge to date of testing ranged between 17 to 26 months (*M* = 22.2, *SD* = 2.1). Based on the NEWS2 categories, 14.6% of the sample experienced mild, 43.9% experienced medium, and 41.5% had a history of high COVID-19 clinical severity.

Due to interruption in testing procedures, one patient did not complete the PHQ-9, GAD-7 and PSQI and 2 patients did not complete any of the cognitive tests. Two additional participants did not complete the OTMT, and 4 patients’ WAIS-IV Digit Span data were removed due to evidence of cheating. A complete-case analysis approach was utilized. As such, participants with missing data were not included in relevant tests.

### Characterization of brain fog and associated variables

**Fig 1:**
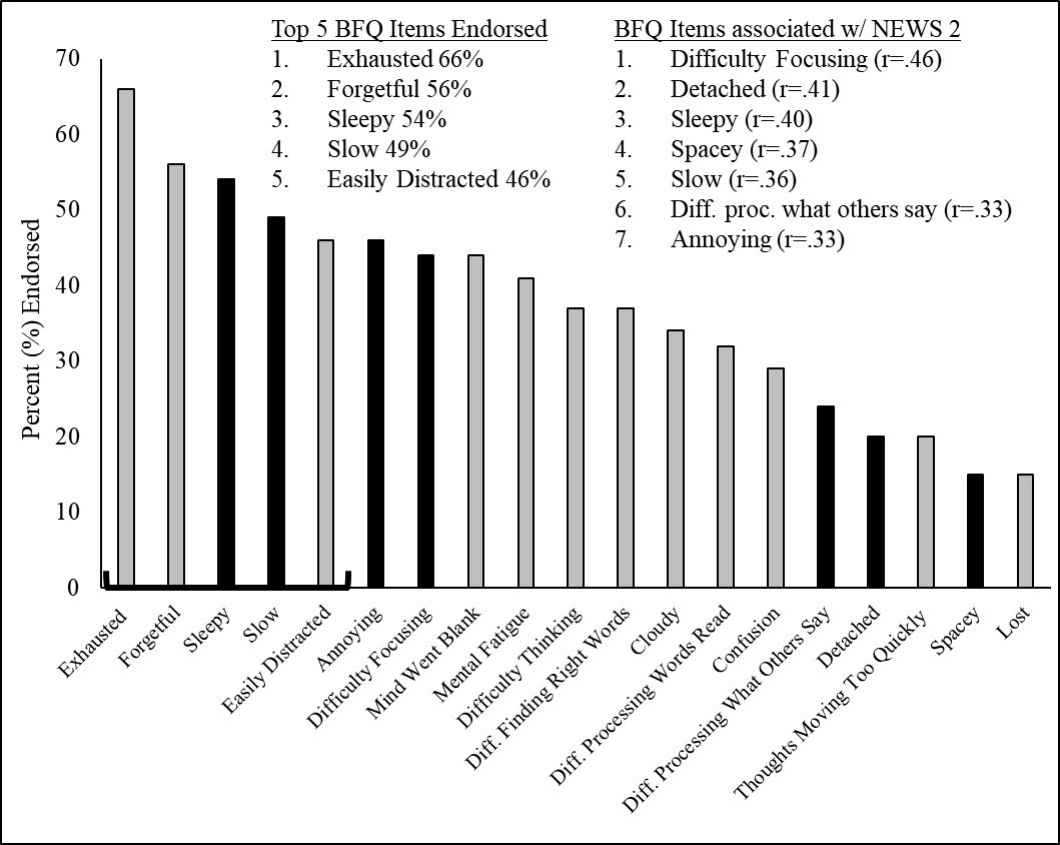
Bar graph representing proportion of sample endorsement for individual Brain Fog Questionnaire (BFQ) items also highlighting items (in black) significantly correlated with COVID-19 clinical severity (NEWS2).

On average, patients endorsed approximately seven of the possible 19 BFQ items (BFQ Total *M* = 7.1, *SD* = 5.3) and 87.8% of the sample endorsed at least one item. The prevalence of each BFQ item ranged from 65.9% to 14.6% (see Table 2) with a gradual linear (vs. stepwise) decline in prevalence when ordered from highest to lowest (see Fig 1). The top 5 BFQ items included exhausted, forgetful, sleepy, slow, and easily distracted. Pearson’s *r* correlations revealed the items most strongly associated with COVID-19 clinical severity (NEWS2) included difficulty focusing, detached, sleepy, spacey, slow, difficulty processing what others say, and annoying. Most BFQ items were associated with depression (PHQ-9) and sleep disturbance (PSQI), while relatively fewer were significantly associated with anxiety (GAD-7) (see Table 2).

**Table 2:** Brain Fog Questionnaire (BFQ) item descriptive statistics and Pearson’s r correlation coefficients for association between BFQ item frequency and COVID-19 severity (NEWS2), depression (PHQ-9), anxiety (GAD 7) and sleep disturbance (PSQI).

	Descriptive			Pearson’s r correlations with Brain Fog Item (Frequency)							
	Endorsed	Frequency	Severity	NEWS 2 (*df* = 41)		PHQ-9 (*df* = 40)		GAD-7 (*df* = 40)		PSQI (*df* = 40)	
	*n*(%)	*M*(*SD*)	*M*(*SD*)	*r*	95% CI	*r*	95% CI	*r*	95% CI	*r*	95% CI
Exhausted	27(65.9)	2.63(2.23)	1.1(1.02)	0.23	[-0.08, 0.50]	0.44	[0.15, 0.66]	0.27	[-0.05, 0.54]	0.47	[0.18, 0.68]
Forgetful	23(56.1)	1.95(2.06)	0.93(0.96)	0.20	[-0.11, 0.48]	0.63	[0.40, 0.79]	0.34	[0.04, 0.59]	0.33	[0.02, 0.58]
Sleepy	22(53.7)	2.07(2.25)	0.83(0.92)	0.40	[0.11, 0.63]	0.30	[-0.01, 0.56]	0.03	[-0.29, 0.34]	0.33	[0.02, 0.58]
Slow	20(48.8)	1.61(1.91)	0.66(0.76)	0.36	[0.05, 0.60]	0.49	[0.21, 0.70]	0.12	[-0.20, 0.41]	0.27	[-0.05, 0.54]
Easily Distracted	19(46.3)	1.34(1.73)	0.63(0.77)	0.17	[-0.14, 0.46]	0.33	[0.02, 0.58]	0.07	[-0.25, 0.37]	0.41	[0.11, 0.64]
Annoying	19(46.3)	1.41(1.77)	0.71(0.90)	0.33	[0.02, 0.58]	0.42	[0.12, 0.64]	0.28	[-0.03, 0.55]	0.35	[0.04, 0.59]
Difficulty Focusing	18(43.9)	1.37(1.87)	0.68(0.85)	0.46	[0.18, 0.67]	0.56	[0.30, 0.74]	0.26	[-0.06, 0.52]	0.47	[0.19, 0.69]
Mind Went Blank	18(43.9)	1.39(1.93)	0.56(0.74)	0.27	[-0.04, 0.53]	0.45	[0.16, 0.67]	0.33	[0.03, 0.59]	0.39	[0.09, 0.63]
Mental Fatigue	17(41.5)	1.37(1.67)	0.66(0.88)	0.09	[-0.22, 0.39]	0.47	[0.18, 0.68]	0.40	[0.10, 0.63]	0.43	[0.14, 0.66]
Difficulty Thinking	15(36.6)	1.22(1.75)	0.59(0.84)	0.08	[-0.24, 0.38]	0.47	[0.19, 0.68]	0.29	[-0.03, 0.55]	0.34	[0.03, 0.59]
Diff. Finding Right Words	15(36.6)	1.07(1.69)	0.54(0.81)	0.31	[0.00, 0.56]	0.40	[0.10, 0.63]	0.04	[-0.27, 0.35]	0.42	[0.12, 0.64]
Cloudy	14(34.1)	1.12(1.75)	0.51(0.78)	0.26	[-0.05, 0.53]	0.47	[0.19, 0.69]	0.19	[-0.13, 0.47]	0.52	[0.25, 0.72]
Diff. Processing Words Read	13(31.7)	1.07(1.85)	0.46(0.78)	0.08	[-0.24, 0.38]	0.38	[0.08, 0.62]	0.16	[-0.16, 0.45]	0.40	[0.10, 0.63]
Confusion	12(29.3)	0.88(1.42)	0.44(0.74)	0.15	[-0.17, 0.44]	0.52	[0.25, 0.72]	0.37	[0.07, 0.61]	0.47	[0.18, 0.68]
Diff. Processing What Others Say	10(24.4)	0.63(1.16)	0.32(0.61)	0.33	[0.03, 0.58]	0.54	[0.28, 0.73]	0.06	[-0.26, 0.36]	0.52	[0.25, 0.72]
Detached	8(19.5)	0.41(1.16)	0.24(0.58)	0.41	[0.12, 0.64]	0.53	[0.26, 0.72]	0.18	[-0.14, 0.47]	0.40	[0.10, 0.63]
Thoughts Moving Too Quickly	8(19.5)	0.61(1.30)	0.29(0.68)	0.03	[-0.28, 0.34]	0.36	[0.05, 0.60]	0.42	[0.13, 0.65]	0.43	[0.14, 0.66]
Spacey	6(14.6)	0.39(1.00)	0.29(0.78)	0.37	[0.07, 0.61]	0.23	[-0.09, 0.50]	0.11	[-0.21, 0.41]	0.38	[0.07, 0.62]
Lost	6(14.6)	0.37(0.94)	0.24(0.62)	-0.21	[-0.49, 0.1]	0.16	[-0.17, 0.45]	0.35	[0.04, 0.60]	0.11	[-0.21, 0.41]

Brain fog total scores were significantly associated with depression, anxiety, sleep disturbance, and COVID-19 clinical severity (see Table 3). Brain fog was not associated with age or number of comorbidities. The only cognitive measure associated with BFQ total score was WAIS-IV digit span backward; however, this effect was non-significant after controlling for depression (partial correlation *r* = -.12, *p* = .49).

**Table 3:** *Results of (A) Pearson’s r correlations and (B) independent sample t-tests among measures of COVID-19 clinical severity (NEWS2)*, *depression (PHQ-9)*, *anxiety (GAD 7)*, *sleep disturbance (PSQI)*, *brain fog (BFQ)*, *and demographic variables (i*.*e*., *Age*, *Comorbidities*, *Sex*, *and Ethnicity) and history of inpatient respiratory distress (ARDS/AHRF vs*. *SOB/none)*.

A.	Pearson’s r correlation														
	**NEWS 2**			**PHQ-9**			**GAD-7**			**PSQI**			**BFQ Total**		
	**r (df = 41)**	**95% CI**		**r (df = 40)**	**95% CI**		**r (df = 40)**	**95% CI**		**r (df = 40)**	**95% CI**		**r (df = 41)**	**95% CI**	
**NEWS 2**	6.5(2.7)														
**PHQ-9**	0.2	[-0.12, 0.48]		8.0(6.4)											
**GAD-7**	-0.13	[-0.43, 0.19]		0.64	[0.4, 0.79]		5.1(4.4)								
**PSQI**	0.2	[-0.12, 0.48]		0.62	[0.38, 0.78]		0.36	[0.05, 0.6]		8.8(4.5)					
**BFQ Total**	0.34	[0.04, 0.59]		0.67	[0.45, 0.81]		0.35	[0.04, 0.59]		0.72	[0.53, 0.84]		7.1(5.3)		
**Age**	-0.02	[-0.33, 0.29]		-0.23	[-0.51, 0.08]		-0.25	[-0.52, 0.06]		-0.05	[-0.36, 0.26]		-0.14	[-0.43, 0.17]	
**Comorbidities**	-0.08	[-0.38, 0.23]		0.04	[-0.28, 0.35]		-0.02	[-0.33, 0.29]		0.44	[0.15, 0.66]		0.05	[-0.27, 0.35]	
**B.**	**Independent sample t-tests**														
	**t(df)**	**d**	**95% CI**	**t(df)**	**d**	**95% CI**	**t(df)**	**d**	**95% CI**	**t(df)**	**d**	**95% CI**	**t(df)**	**d**	**95% CI**
**Sex**	-2.18(39)	-0.68	[-1.31, -0.05]	-0.95(32.53)*	-0.31	[-0.93, 0.32]	1.79(38)	0.57	[-0.07, 1.2]	-1.15(38)	-0.36	[-0.99, 0.26]	-1.74(39)	-0.54	[-1.17, 0.09]
**Ethnicity**	-0.27(39)	-0.09	[-0.79, 0.60]	-1.16(24.79)*	-0.34	[-1.06, 0.38]	0.02(38)	0.01	[-0.71, 0.72]	-1.68(38)	-0.61	[-1.34, 0.12]	-1.84(39)	-0.65	[-1.35, 0.06]
**Resp. Distress**	2.92(39)	-0.92	[-1.56, -0.27]	0.17(38)	-0.05	[-0.68, 0.57]	-0.05(38)	0.02	[-0.61, 0.64]	-0.56(38)	0.18	[-0.45, 0.81]	0.14(39)	-0.04	[-0.66, 0.57]

*Levene’s Test for Equality of Variances was statistically significant (*p* < .05).

*Note*. Cross-diagonal of correlation matrix represent measure descriptive statistics (*M* & *SD*)

### Cognitive assessments, psychiatric measures, and associated variables

**Fig 2:**
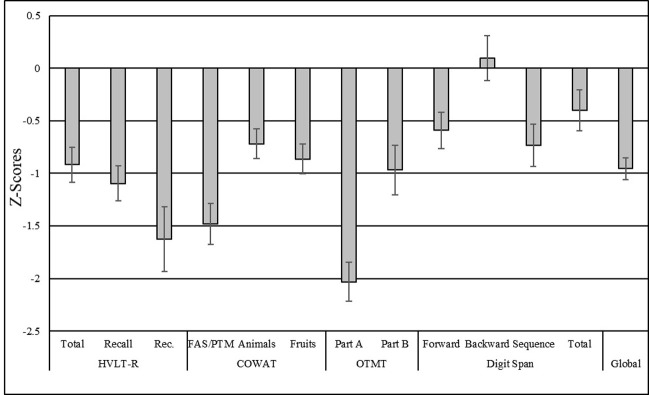
Bar graph illustrating descriptive statistics (*M* and *S*.*E*.) for all cognitive measures.

On average, patients in the sample performed approximately 1 standard deviation below the mean, as determined by respective normative samples (see Table 4). The greatest deficits were evidenced on tasks of verbal memory recognition (HVLT-R Recognition), letter fluency (COWAT FAS/PTM), and processing speed (OTMT Part A). Patients’ performance was relatively better on measures of attention and working memory (WAIS-IV digit span total), particularly on digit span backward. See Fig 2 for graphic illustration of standardized (Z-score) averages for all cognitive tests.

**Table 4:** Cognitive measure descriptive statistics, independent sample t-tests evaluating group differences in sex (female vs. male), ethnicity (Hispanic vs. Non-Hispanic), and respiratory distress (ARDS/AHRF vs. SOB/None), as well as Pearson’s r correlations testing associations with COVID-19 clinical severity (NEWS2), depression (PHQ-9), anxiety (GAD 7), sleep disturbance (PSQI), and brain fog (BFQ) total scores.

	**HVLT-R**											
	**Total**			**Recall**			**Recognition**					
**M(SD)**	-0.92(1.05)			-1.09(1.04)			-1.63(1.91)					
**t-tests (df)**	**t(37)**	**d**	**95%CI**	**t(37)**		**95%CI**	**t(37)**		**95%CI**			
**Sex**	-0.68	-0.22	[-0.85, 0.42]	0.26	0.08	[-0.55, 0.71]	-1.39	-0.45	[-1.08, 0.19]			
**Ethnicity**	-1.34	-0.51	[-1.26, 0.25]	-1.50	-0.57	[-1.32, 0.19]	0.43	0.16	[-0.58, 0.91]			
**Resp. Distress**	0.74	0.24	[-0.4, 0.88]	-0.04	-0.01	[-0.65, 0.63]	1.15	0.37	[-0.27, 1.02]			
**Pearson’s r (N)**	**r(39)**	**95%CI**		**r(39)**	**95%CI**		**r(39)**	**95%CI**				
**NEWS 2**	-0.21	[-0.49, 0.12]		-0.17	[-0.46, 0.15]		-0.21	[-0.49, 0.12]				
**PHQ-9**	0.01	[-0.31, 0.33]		-0.11	[-0.41, 0.21]		0.07	[-0.25, 0.38]				
**GAD 7**	-0.11	[-0.41, 0.21]		-0.14	[-0.44, 0.18]		0.03	[-0.29, 0.34]				
**PSQI**	-0.03	[-0.34, 0.29]		-0.11	[-0.41, 0.22]		-0.08	[-0.39, 0.24]				
**BFQ Total**	0.01	[-0.31, 0.32]		-0.04	[-0.35, 0.28]		0.02	[-0.3, 0.33]				
	**COWAT**											
	**FAS/PTM**			**Animals**			**Fruits**					
**M(SD)**	-1.48(1.21)			-0.72(0.86)			-0.86(0.87)					
**t-tests (df)**	**t(37)**		**95%CI**	**t(37)**		**95%CI**	**t(37)**		**95%CI**			
**Sex**	0.80	0.26	[-0.38, 0.89]	-0.25	-0.08	[-0.71, 0.55]	0.07	0.02	[-0.61, 0.65]			
**Ethnicity**	2.29	0.87	[0.09, 1.63]	-0.10	-0.04	[-0.78, 0.71]	1.98	0.75	[-0.02, 1.51]			
**Resp. Distress**	1.58	0.51	[-0.14, 1.16]	-0.36	-0.12	[-0.75, 0.52]	1.80	0.59	[-0.07, 1.23]			
**Pearson’s r (N)**	**r(39)**	**95%CI**		**r(39)**	**95%CI**		**r(39)**	**95%CI**				
**NEWS 2**	-0.13	[-0.43, 0.19]		-0.11	[-0.41, 0.21]		-0.21	[-0.49, 0.11]				
**PHQ-9**	-0.23	[-0.51, 0.09]		0.01	[-0.3, 0.33]		0.18	[-0.14, 0.47]				
**GAD 7**	0.06	[-0.26, 0.37]		0.002	[-0.32, 0.32]		0.30	[-0.02, 0.56]				
**PSQI**	-0.19	[-0.48, 0.13]		0.05	[-0.27, 0.36]		-0.12	[-0.42, 0.2]				
**BFQ Total**	-0.18	[-0.47, 0.14]		-0.002	[-0.32, 0.31]		0.18	[-0.15, 0.47]				
	**WAIS-IV Digit Span**											
	**Forward**			**Backward**			**Sequence**			**Total**		
**M(SD)**	-0.59(1.02)			0.10(1.25)			-0.73(1.2)			-0.4(1.14)		
**t-tests (df)**	**t(33)**	**d**	**95%CI**	**t(33)**	**d**	**95%CI**	**t(33)**	**d**	**95%CI**	**t(33)**	**d**	**95%CI**
**Sex**	0.96	0.33	[-0.35, 0.99]	0.32	0.11	[-0.56, 0.77]	-0.2	-0.07	[-0.73, 0.6]	0.57	0.19	[-0.48, 0.86]
**Ethnicity**	0.60	0.25	[-0.58, 1.08]	1.02	0.43	[-0.41, 1.26]	0.98	0.42	[-0.42, 1.25]	1.04	0.44	[-0.4, 1.27]
**Resp. Distress**	< .01	< .01	[-0.69, 0.69]	2.19	0.77	[0.05, 1.47]	1.54	0.54	[-0.16, 1.23]	1.52	0.53	[-0.17, 1.23]
**Pearson’s r (N)**	**r(35)**	**95%CI**		**r(35)**	**95%CI**		**r(35)**	**95%CI**		**r(35)**	**95%CI**	
**NEWS 2**	-0.01	[-0.34, 0.33]		-0.41	[-0.66, -0.09]		-0.16	[-0.47, 0.18]		-0.28	[-0.56, 0.05]	
**PHQ-9**	-0.27	[-0.56, 0.07]		-0.51	[-0.72, -0.21]		-0.31	[-0.59, 0.02]		-0.46	[-0.69, -0.15]	
**GAD 7**	-0.08	[-0.4, 0.26]		-0.24	[-0.53, 0.1]		-0.27	[-0.56, 0.07]		-0.26	[-0.55, 0.08]	
**PSQI**	-0.02	[-0.35, 0.32]		-0.30	[-0.58, 0.03]		-0.20	[-0.5, 0.14]		-0.27	[-0.56, 0.07]	
**BFQ Total**	-0.09	[-0.41, 0.25]		-0.44	[-0.68, -0.13]		-0.18	[-0.48, 0.17]		-0.31	[-0.58, 0.03]	
	**OTMT**									**Global**		
	**Part A**			**Part B**								
**M(SD)**	-2.03(1.13)			-0.97(1.44)						-0.96(0.66)		
**t-tests (df)**	**t(35)**	**d**	**95%CI**	**t(35)**	**d**	**95%CI**				**t(37)**	**d**	**95%CI**
**Sex**	2.51	0.83	[0.15, 1.5]	0.59	0.20	[-0.45, 0.84]				0.46	0.15	[-0.48, 0.77]
**Ethnicity**	0.46	0.18	[-0.58, 0.93]	5.98*	1.39	[0.56, 2.2]				1.52	0.58	[-0.18, 1.33]
**Resp. Distress**	-0.78	-0.26	[-0.91, 0.4]	2.52	0.84	[0.15, 1.51]				1.99	0.65	[-0.01, 1.3]
**Pearson’s r (N)**	**r(37)**	**95%CI**		**r(37)**	**95%CI**					**r(39)**	**95%CI**	
**NEWS 2**	-0.03	[-0.35, 0.3]		-0.22	[-0.51, 0.11]					-0.36	[-0.61, -0.05]	
**PHQ-9**	-0.11	[-0.42, 0.22]		-0.22	[-0.51, 0.11]					-0.31	[-0.57, 0.01]	
**GAD 7**	0.002	[-0.33, 0.32]		-0.08	[-0.39, 0.25]					-0.13	[-0.43, 0.19]	
**PSQI**	-0.32	[-0.58, 0.01]		-0.20	[-0.49, 0.13]					-0.28	[-0.55, 0.04]	
**BFQ Total**	-0.17	[-0.47, 0.16]		-0.10	[-0.41, 0.23]					-0.21	[-0.49, 0.12]	

*Levene’s Test for Equality of Variances was statistically significant (p < .05)

Demographic group differences indicated that females were slower than males on OTMT Part A. These differences remained significant per follow-up analyses of covariance (ANCOVA), testing group differences in sex while controlling for ethnicity, time since discharge and total comorbidities (*F* (1, 32) = 6.51, *p* = .02, partial eta-squared (*η*_*p*_^*2*^) = .17). Group differences based on ethnicity were also evidenced indicated that Hispanics performed worse than non-Hispanics on tests of letter fluency (COWAT FAS/PTM) and speeded executive set-shifting (OTMT Trails B).. These effects also remained significant in follow-up ANCOVA analyses controlling for sex, time since discharge and total comorbidities (FAS/PTM: *F*(1, 34) = 4.64, *p* = .04, *η*_*p*_^*2*^ = .12; OTMT Trails B: *F*(1, 32) = 11.66, *p* = .002, *η*_*p*_^*2*^ = .27).

On average, reports of both depression and anxiety fell within the mild range (see Table 3). Thirty-five percent of the sample reported symptoms consistent with a moderate level of depression or higher, while 18% of the patients did so for anxiety. On the PSQI, 75% of the sample reported symptoms indicating elevated sleep disturbance.

Sleep disturbance was positively associated with total number of comorbidities, but not with age, sex, or ethnicity. Depression and anxiety were not found to significantly correlate with these measures (see Table 3).

### Associations with COVID-19 severity

**Fig 3:**
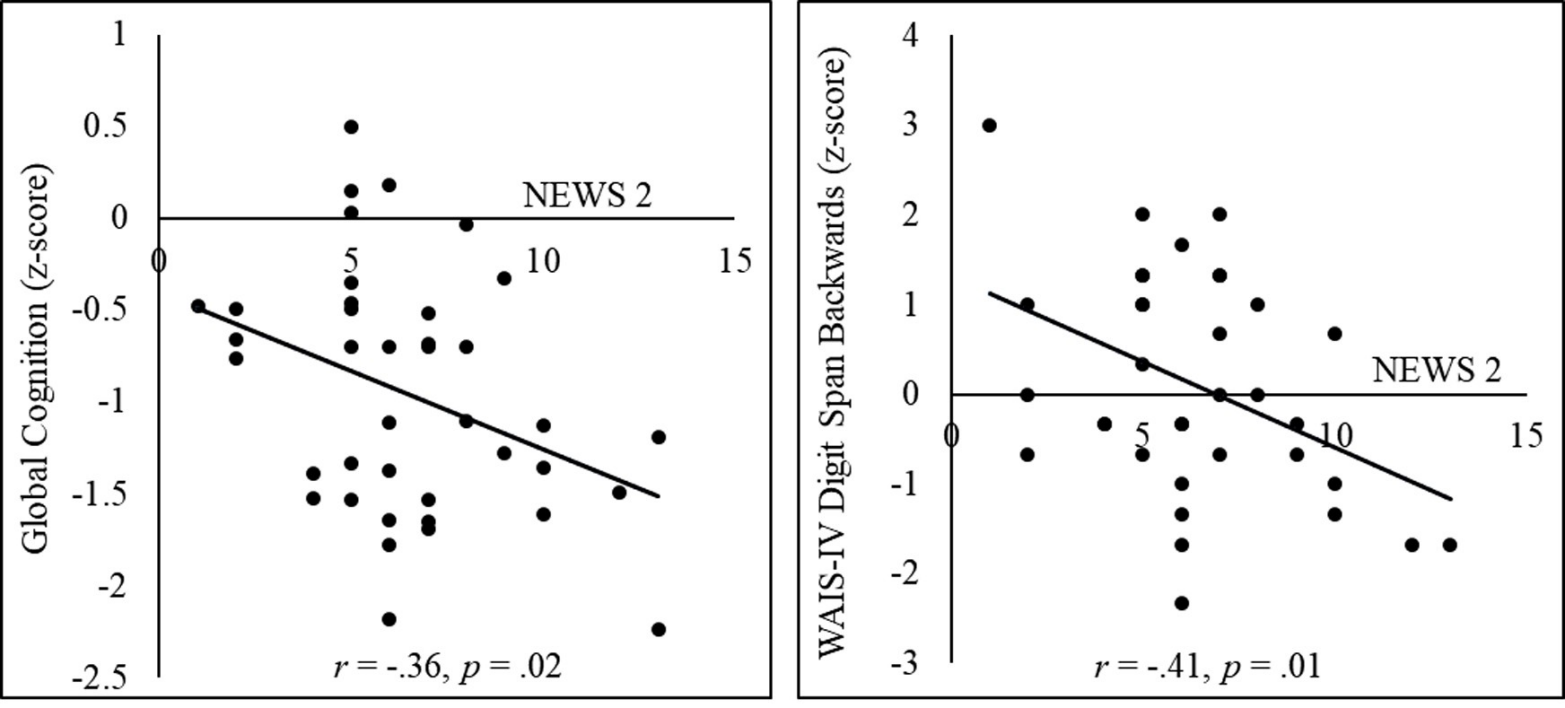
Scatter plots representing associations between COVID-19 clinical severity (NEWS 2) and cognitive measures of global cognition and working memory (WAIS-IV digits backward).

See Table 4 for results of all independent sample *t*-tests and Pearson’s *r* correlations among cognitive tests. COVID-19 clinical severity, as indexed by NEWS2, was significantly associated with WAIS-IV digit span backward and global cognition (see Fig 3). Follow-up multiple regression analyses indicated these findings remained after controlling for ethnicity, sex, time since discharge and total comorbidities (WAIS-IV digit span backward: *β* = -.43, *t* = -2.45, *p* = .02; global cognition: *β* = -.38, *t* = -2.30, *p* = .03).

**Fig 4:**
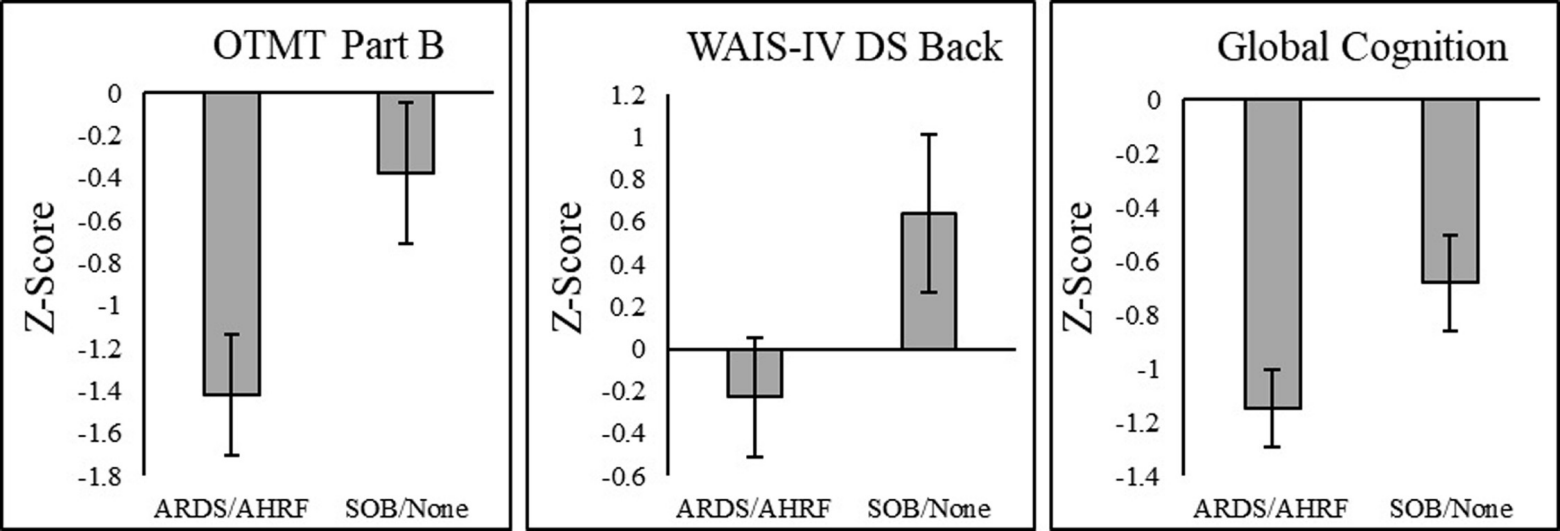
Bar graphs illustrate significant group differences in cognitive performance based on inpatient respiratory distress (ARDS/AHRF vs. SOB/None). Bars depict estimated marginal means controlling for total comorbidities and demographic variables of sex and ethnicity. Error bars represent standard errors from the estimated marginal means.

Similarly, independent sample *t*-tests looking at differences based on diagnosed respiratory distress (i.e., ARDS/AHRF vs. SOB/None) revealed that those diagnosed with respiratory distress performed worse on WAIS-IV digit span backward and OTMT Part B. These group differences were also marginally significant for global cognition, indicating that those with a history of ARDS/AHRF (*M* = -1.13, *SD* = 0.56) were somewhat worse than those with history of SOB/None (*M* = -0.71, *SD* = 0.74), (*t*(37) = 1.99, *p* = .05, *d* = .65). Follow-up ANCOVAs controlling for sex, ethnicity, time since discharge and total comorbidities demonstrated similar findings (WAIS-IV digit span backward: *F*(1, 29) = 3.09, *p* = .009, *η*_*p*_^*2*^ = .10; OTMT Part B: *F*(1, 31) = 4.98, *p* = .03, *η*_*p*_^*2*^ = .14; global cognition: *F*(1, 33) = 3.71, *p* = .06, *η*_*p*_^*2*^ = .010). See Fig 4 for bar graphs demonstrating these group differences. No significant associations or group differences were found between all psychiatric measures and either NEWS2 or respiratory distress groups, respectively (see Table 3).

## Discussion

In this study, we describe brain fog, cognitive, and psychiatric sequalae, in a racially and ethnically diverse cohort of adults with a history of hospitalization due to COVID-19. Our findings highlight how prevalent brain fog symptoms such as exhaustion, forgetfulness and slowed processing are in previously hospitalized PASC patients, and how brain fog is associated with depression, sleep disturbances and anxiety. Cognitive deficits were also apparent, especially in areas of verbal memory, letter fluency and processing speed. COVID-19 clinical severity was linked to worse cognitive outcomes, particularly in global cognition and working memory.

Walgren et al’s population based longitudinal study in Sweden found that 84% of patients reported persistent symptoms at 24 months, with the most common being mental fatigue (65%), difficulty remembering (60%), weakness (58%) and word finding difficulties (40%) [6]. The results showed that most patients experienced persistent sequalae and impaired quality of life, with fatigue being the most cited. They also found that women and individuals with pre-existing conditions were more likely to have ongoing problems. Our results also demonstrated a high prevalence of symptoms, with patients endorsing 7 on average. Those who had greater COVID-19 clinical severity (as indexed by the NEWS2) endorsed more brain fog symptoms overall. Our findings showed that brain fog complaints may not reflect objective cognitive findings. This emphasizes the value of objective testing of perceived deficits. Our results also reinforce Walgren et al’s conclusions that some post-acute COVID-19 survivors experience persistent subjective cognitive and affective symptoms two years after infection [8]. We also found that brain fog total score was significantly associated with depression, anxiety, and sleep disturbance. In their original BFQ paper, Ross et al reported that sleep disturbances (particularly the presence of comorbid sleep disorders) may affect brain fog as well as negatively impacting cognitive performance [25]. Thereby, it is also likely that similar to findings by Graham et al [39], a subset of patients reporting subjective complaints may be affected by other psychiatric features factoring into their brain fog symptoms. These findings highlight the need for continued monitoring and support for patients with post-COVID-19 condition, particularly those with pre-existing conditions and women.

We also investigated cognitive performance and psychiatric symptoms and whether these variables correspond with demographic and self-report measures. For the cognitive assessments, we standardized the scores using normative reference groups. On average, patients exhibited poor cognitive performance compared to the normative samples. Consistent with brain fog reports of word finding difficulties and feeling forgetful, test scores on the Controlled Oral Word Association Test (COWAT) letters and the HVLT-R recognition were notably deficient, implying difficulties with phonemic fluency and memory. In line with brain fog complaints of slowness and suggestive of poor processing speed, scores for the Oral Trails A were also deficient. Demographic differences were observed, with females scoring poorly on processing speed compared to males and Hispanics exhibiting poorer performance on phonemic fluency, semantic fluency and executive set shifting than non-Hispanics. The extent to which previous acute COVID-19 infection contributes to these differences is unknown, however it is possible that other underlying factors might be involved such as cultural or language differences. Hispanics also experience a high prevalence of comorbidities such as type 2 diabetes, which has been linked to an increased risk of cognitive decline [40]. Additionally, psychosocial, and socioeconomic factors such as lower educational attainment and lower income may be contributing factors [41].

We also examined the influence of COVID-19 clinical severity on cognitive and psychiatric outcomes. COVID-19 clinical severity was significantly associated with poorer performance on digit span backward and global cognition. Individuals who experienced acute hypoxic respiratory failure (AHRF) or acute respiratory distress syndrome (ARDS) while hospitalized performed significantly worse on working memory and executive set shifting tasks and had marginally worse global cognition. These findings corroborate earlier research reporting executive dysfunction in PASC patients [42]. It is important to consider that 35% of our sample met criteria for moderate to severe depression, thus we cannot disentangle these findings from previous research reporting executive dysfunction due to depression [43]. There may be other etiological factors contributing to the neuropsychological symptoms of PASC. One prominent hypothesis is that infection induced neuroinflammation and hypoxic-ischemic tissue damage may play a role in the presentation of these features [44].

This study has multiple limitations, including a small sample size and the lack of control group. While we were able to assess the cognitive and psychiatric symptoms, we do not know if what was captured is indeed due to prior COVID-19 infection or other factors (i.e., post intensive care syndrome, social isolation, socioeconomic stressors, etc.) While the initial pool of potential participants was 282, 41 patients could be reached and agreed to participate, introducing a possible selection bias. Ross et al acknowledge that their BFQ demonstrated validity in a postural orthostatic tachycardia syndrome sample [25]. However, our study is the first known use of the BFQ in COVID-19 patients, thus further research is needed to assess its validity in this new population. We back translated the BFQ for use with Spanish speaking patients, which is a novelty of this study considering the lack of research in this demographic. It is worth noting that the validity of the BFQ in Spanish has yet to be established. In terms of battery administration, employing face to face testing or HIPAA compliant visual telehealth methods, rather than phone, would provide better controlled testing conditions. While there were issues with completing the battery over the telephone, it is a strength in that we were able to reach patients who may not come to a research site for assessment due to health-related disabilities, mobility, or transportation issues.

## Conclusion

The study provides data on the post-acute long-term cognitive and psychiatric sequalae in underrepresented ethnic and racial groups. It underscores the prevalence of brain fog in post COVID-19 patients and its relationship to cognition, depression, anxiety, sleep, demographics, and COVID-19 severity.

## Supporting information

S1 File Information on self-report psychiatric measures administered to participants, including the PHQ-9, GAD-7 and PSQI. (DOCX)

## References

[1] Hingorani KS, Bhadola S, Cervantes-Arslanian AM. COVID-19 and the brain. Trends in cardiovascular medicine. 2022;32(6):323–30. doi: 10.1016/j.tcm.2022.04.004 35461991 PMC9022395

[2] Nalbandian A, Sehgal K, Gupta A, Madhavan MV, McGroder C, Stevens JS, et al. Post-acute COVID-19 syndrome. Nature medicine. 2021 Apr;27(4):601–15. doi: 10.1038/s41591-021-01283-z 33753937 PMC8893149

[3] Yang C, Zhao H, Shannon CP, Tebbutt SJ. Omicron variants of SARS-CoV-2 and long COVID. Frontiers in Immunology. 2022;13:1061686. doi: 10.3389/fimmu.2022.1061686 36569883 PMC9780375

[4] Takao M, Ohira M. Neurological post‐acute sequelae of SARS‐CoV‐2 infection. Psychiatry and Clinical Neurosciences. 2023;77(2):72–83. doi: 10.1111/pcn.13481 36148558 PMC9538807

[5] Miskowiak KW, Johnsen S, Sattler SM, Nielsen S, Kunalan K, Rungby J, et al. Cognitive impairments four months after COVID-19 hospital discharge: Pattern, severity and association with illness variables. European Neuropsychopharmacology. 2021;46:39–48. doi: 10.1016/j.euroneuro.2021.03.019 33823427 PMC8006192

[6] Poletti S, Palladini M, Mazza MG, De Lorenzo R, Furlan R, Ciceri F, et al. Long-term consequences of COVID-19 on cognitive functioning up to 6 months after discharge: role of depression and impact on quality of life. European archives of psychiatry and clinical neuroscience. 2021:1–0. doi: 10.1007/s00406-021-01346-9 34698871 PMC8546751

[7] Ferrucci R, Dini M, Rosci C, Capozza A, Groppo E, Reitano MR, et al. One‐year cognitive follow‐up of COVID‐19 hospitalized patients. European Journal of Neurology. 2022;29(7):2006–14. doi: 10.1111/ene.15324 35285122 PMC9111730

[8] Wahlgren C, Forsberg G, Divanoglou A, Balkhed ÅÖ, Niward K, Berg S, et al. Two-year follow-up of patients with post-COVID-19 condition in Sweden: A prospective cohort study. The Lancet Regional Health–Europe. 2023;28. doi: 10.1016/j.lanepe.2023.100595 36855599 PMC9951394

[9] Rogers J. P., Chesney E., Oliver D., Pollak T. A., McGuire P., Fusar-Poli P., et al. (2020). Psychiatric and neuropsychiatric presentations associated with severe coronavirus infections: a systematic review and meta-analysis with comparison to the COVID-19 pandemic. The Lancet Psychiatry, 7(7), 611–627. doi: 10.1016/S2215-0366(20)30203-0 32437679 PMC7234781

[10] Taquet M, Geddes JR, Husain M, Luciano S, Harrison PJ. 6-month neurological and psychiatric outcomes in 236 379 survivors of COVID-19: a retrospective cohort study using electronic health records. The Lancet Psychiatry. 2021;8(5):416–27. doi: 10.1016/S2215-0366(21)00084-5 33836148 PMC8023694

[11] Ceban F, Ling S, Lui LM, Lee Y, Gill H, Teopiz KM, et al. Fatigue and cognitive impairment in Post-COVID-19 Syndrome: A systematic review and meta-analysis. Brain, behavior, and immunity. 2022;101:93–135. doi: 10.1016/j.bbi.2021.12.020 34973396 PMC8715665

[12] Nordvig A, Noble J. Post-COVID Brain Fog: A patient registry and cross-disciplinary approach to characterization, treatment, and etiology. (4288). Neurology. 2021.http://n.neurology.org/content/96/15_Supplement/4288.abstract

[13] Ferrarese C, Silani V, Priori A, Galimberti S, Agostoni E, Monaco S, et al, Italian Society of Neurology (SIN). An Italian multicenter retrospective-prospective observational study on neurological manifestations of COVID-19 (NEUROCOVID). Neurological Sciences. 2020;41:1355–9. doi: 10.1007/s10072-020-04450-1 32430621 PMC7235538

[14] Jacobs MM, Evans E, Ellis C. Racial, ethnic, and sex disparities in the incidence and cognitive symptomology of long COVID-19. Journal of the National Medical Association. 2023;115(2):233–43. doi: 10.1016/j.jnma.2023.01.016 36792456 PMC9923441

[15] Wang C., Ramasamy A., Verduzco-Gutierrez M., Brode W. M., & Melamed E. (2023). Acute and post-acute sequelae of SARS-CoV-2 infection: a review of risk factors and social determinants. Virology Journal, 20(1), 124. doi: 10.1186/s12985-023-02061-8 37328773 PMC10276420

[16] CDC Covid Data tracker [Internet]. Centers for Disease Control and Prevention; [cited 2023 Sept 7]. Available from: https://covid.cdc.gov/covid-data-tracker/#demographics

[17] Chen AK, Wang X, McCluskey LP, Morgan JC, Switzer JA, Mehta R, et al. Neuropsychiatric sequelae of long COVID-19: Pilot results from the COVID-19 neurological and molecular prospective cohort study in Georgia, USA. Brain, Behavior, & Immunity-Health. 2022;24:100491. doi: 10.1016/j.bbih.2022.100491 35873350 PMC9290328

[18] Bai F, Tomasoni D, Falcinella C, Barbanotti D, Castoldi R, Mulè G, et al. Female gender is associated with long COVID syndrome: a prospective cohort study. Clinical Microbiology and Infection. 2022;28(4):611–e9. doi: 10.1016/j.cmi.2021.11.002 34763058 PMC8575536

[19] Owens CD, Pertuz GM, Sanchez JC, Ayala J, Pimentel LH, Lamb C, et al. The COVID-19 Pandemic in a Hispanic population: A primary care perspective. The Journal of the American Board of Family Medicine. 2022;35(4):686–94. doi: 10.3122/jabfm.2022.04.210163 35896459

[20] Deloitte. Why improving inclusion and diversity in clinical trials should be a research priority [Internet]. 2020 [cited 2023 Sept 7]. Available from: https://www2.deloitte.com/us/en/blog/health-care-blog/2019/why-improving-inclusion-and-diversity-in-clinical-trials-should-be-a-research-priority.html

[21] Sobczak E, Swafford EP, Samano D, Bass D, Ghamasaee P, Kottapally M, et al. Posttraumatic Stress Symptoms Among COVID-19 Survivors After Hospitalization. The Journal of Neuropsychiatry and Clinical Neurosciences. 2023:appi-neuropsych. doi: 10.1176/appi.neuropsych.20220126 36710628

[22] Kroenke K, Spitzer RL, Williams JB. The PHQ‐9: validity of a brief depression severity measure. Journal of General Internal Medicine. 2001;16(9):606–13. doi: 10.1046/j.1525-1497.2001.016009606.x 11556941 PMC1495268

[23] Spitzer RL, Kroenke K, Williams JB, Löwe B. A brief measure for assessing generalized anxiety disorder: the GAD-7. Archives of Internal Medicine. 2006;166(10):1092–7. doi: 10.1001/archinte.166.10.1092 16717171

[24] Buysse DJ, Reynolds CF III, Monk TH, Hoch CC, Yeager AL, Kupfer DJ. Quantification of subjective sleep quality in healthy elderly men and women using the Pittsburgh Sleep Quality Index (PSQI). Sleep. 1991;14(4):331–8. https://pubmed.ncbi.nlm.nih.gov/1947597/ 1947597

[25] Ross AJ, Medow MS, Rowe PC, Stewart JM. What is brain fog? An evaluation of the symptom in postural tachycardia syndrome. Clinical Autonomic Research. 2013;23:305–11. doi: 10.1007/s10286-013-0212-z 23999934 PMC3896080

[26] Benedict RH, Schretlen D, Groninger L, Brandt J. Hopkins Verbal Learning Test–Revised: Normative data and analysis of inter-form and test-retest reliability. The Clinical Neuropsychologist. 1998;12(1):43–55. https://www.tandfonline.com/doi/abs/10.1076/clin.12.1.43.1726

[27] Arango-Lasprilla JC, Rivera D, Garza MT, Saracho CP, Rodríguez W, Rodríguez-Agudelo Y, et al. Hopkins verbal learning test–revised: Normative data for the Latin American Spanish speaking adult population. NeuroRehabilitation. 2015;37(4):699–718. doi: 10.3233/NRE-151286 26639933

[28] Tombaugh TN, Kozak J, Rees L. Normative data stratified by age and education for two measures of verbal fluency: FAS and animal naming. Archives of Clinical Neuropsychology. 1999;14(2):167–77. https://pubmed.ncbi.nlm.nih.gov/14590600/ 14590600

[29] Rey GJ. Multilingual aphasia examination-spanish development and normative data. University of Houston; 1989. https://www.proquest.com/openview/7d22fae9888152cfab6971329667fa86/1?pq-origsite=gscholar&cbl=18750&diss=y

[30] Olabarrieta-Landa L, Rivera D, Galarza-Del-Angel J, Garza MT, Saracho CP, Rodríguez W, et al. Verbal fluency tests: Normative data for the Latin American Spanish speaking adult population. NeuroRehabilitation. 2015;37(4):515–61. doi: 10.3233/NRE-151279 26639930

[31] Acevedo A, Loewenstein DA, Barker WW, Harwood DG, Luis C, Bravo M, et al. Category fluency test: normative data for English-and Spanish-speaking elderly. Journal of the International Neuropsychological Society. 2000;6(7):760–9. doi: 10.1017/s1355617700677032 11105466

[32] Mrazik M, Millis S, Drane DL. The oral trail making test: effects of age and concurrent validity. Archives of Clinical Neuropsychology. 2010;25(3):236–43. doi: 10.1093/arclin/acq006 20197294 PMC2858599

[33] Wechsler D. Wechsler adult intelligence scale–Fourth Edition (WAIS–IV). San Antonio, TX: NCS Pearson. 2008;22(498):1.

[34] Pontón MO, Satz P, Herrera L, Ortiz F, Urrutia CP, Young R, et al. Normative data stratified by age and education for the Neuropsychological Screening Battery for Hispanics (NeSBHIS): Initial report. Journal of the International Neuropsychological Society. 1996;2(2):96–104. doi: 10.1017/s1355617700000941 9375194

[35] Thor Y, Basche KE, Wahoske ML, Spalitta A, Chin NA, Gleason CE, et al. Feasibility and reliability of telephone‐based cognitive assessment in middle‐aged and older adults in the Wisconsin Alzheimer’s Disease Research Center. Alzheimer’s & Dementia. 2022;18:e061645. https://alz-journals.onlinelibrary.wiley.com/doi/full/10.1002/alz.061645

[36] Parks AC, Davis J, Spresser CD, Stroescu I, Ecklund-Johnson E. Validity of in-home teleneuropsychological testing in the wake of COVID-19. Archives of Clinical Neuropsychology. 2021;36(6):887–96. doi: 10.1093/arclin/acab002 33561190 PMC7929470

[37] Rapp SR, Legault C, Espeland MA, Resnick SM, Hogan PE, Coker LH, et al. Validation of a cognitive assessment battery administered over the telephone. Journal of the American Geriatrics Society. 2012;60(9):1616–23. doi: 10.1111/j.1532-5415.2012.04111.x 22985137 PMC3448122

[38] Rigoni M, Torri E, Nollo G, Delle Donne L, Cozzio S. NEWS2 is a valuable tool for appropriate clinical management of COVID-19 patients. European Journal of Internal Medicine. 2021;85:118–20. doi: 10.1016/j.ejim.2020.11.020 33358535 PMC7751376

[39] Graham EL, Clark JR, Orban ZS, Lim PH, Szymanski AL, Taylor C, et al. Persistent neurologic symptoms and cognitive dysfunction in non‐hospitalized Covid‐19 “long haulers”. Annals of Clinical and Translational Neurology. 2021;8(5):1073–85. doi: 10.1002/acn3.51350 33755344 PMC8108421

[40] González HM, Tarraf W, González KA, Fornage M, Zeng D, Gallo LC, et al. Diabetes, cognitive decline, and mild cognitive impairment among diverse Hispanics/Latinos: Study of Latinos–Investigation of Neurocognitive Aging Results (HCHS/SOL). Diabetes Care. 2020;43(5):1111–7. doi: 10.2337/dc19-1676 32139382 PMC7171942

[41] Zahodne LB, Sharifian N, Kraal AZ, Zaheed AB, Sol K, Morris EP, et al. Socioeconomic and psychosocial mechanisms underlying racial/ethnic disparities in cognition among older adults. Neuropsychology. 2021 ;35(3):265. doi: 10.1037/neu0000720 33970660 PMC8363204

[42] Hall PA, Meng G, Hudson A, Sakib MN, Hitchman SC, MacKillop J, et al. Cognitive function following SARS-CoV-2 infection in a population-representative Canadian sample. Brain, Behavior, & Immunity-health. 2022;21:100454. doi: 10.1016/j.bbih.2022.100454 35340304 PMC8934755

[43] Porter RJ, Bourke C, Gallagher P. Neuropsychological impairment in major depression: its nature, origin and clinical significance. Australian & New Zealand Journal of Psychiatry. 2007;41(2):115–28. doi: 10.1080/00048670601109881 17464689

[44] Cosentino G, Todisco M, Hota N, Della Porta G, Morbini P, Tassorelli C, et al. Neuropathological findings from COVID‐19 patients with neurological symptoms argue against a direct brain invasion of SARS‐CoV‐2: A critical systematic review. European Journal of Neurology. 2021;28(11):3856–65. doi: 10.1111/ene.15045 34339563 PMC8444743

