# Supplementary material for: Long-term brain fog and cognitive impairment in previously hospitalized COVID-19 patients
Source: PLoS One. 2024 Aug 29;19(8):e0309102. doi: 10.1371/journal.pone.0309102 (PMC11361661; doi:10.1371/journal.pone.0309102)
Supplement: S1 File — (DOCX) [file pone.0309102.s001.docx]

**Supplemental Information**

**Self-report psychiatric measures:** The Patient Health Questionnaire-9 (PHQ-9) [22], assesses the severity of depression and consists of 9 items that ask about the presence and severity of common depressive symptoms, such as low mood, loss of interest in activities, and feelings of worthlessness. Scores of 5, 10, 15, and 20 represent cut-off scores for mild, moderate, moderately-severe, and severe levels of depression, respectively. The Generalized Anxiety Disorder-7 (GAD-7) [23], assesses anxiety and consists of 7 items that ask about the presence and severity of common anxiety symptoms, such as feeling nervous, worrying too much, and being unable to relax. Scores of 5, 10 and 15 represent cut-off scores for mild, moderate and severe levels of anxiety, respectively. The Pittsburgh Sleep Quality Index (PSQI) [24], is used to assess the quality of sleep and consists of 19 items that ask about various aspects of sleep, such as sleep latency, sleep duration, and sleep disturbances. Scores of 5 or greater indicate elevated sleep disturbance.
